# Centrality of Pregnancy and Prenatal Attachment in Pregnant Nulliparous After Recent Elective or Therapeutic Abortion

**DOI:** 10.3389/fpsyg.2020.607879

**Published:** 2020-12-03

**Authors:** Martina Smorti, Lucia Ponti, Lucia Bonassi, Elena Cattaneo, Chiara Ionio

**Affiliations:** ^1^Department of Surgical, Medical and Molecular Pathology and Critical Care Medicine, University of Pisa, Pisa, Italy; ^2^Department of Education, Languages, Intercultures, Literatures and Psychology, University of Florence, Firenze, Italy; ^3^Department of Mental Health, Azienda Socio Sanitaria Territoriale (ASST) Bergamo-Est, Seriate, Italy; ^4^Department of Psychology, Catholic University of the Sacred Heart, University of Milan, Milan, Italy

**Keywords:** pregnancy, therapeutic abortion, elective abortion, prenatal attachment, centrality of pregnancy

## Abstract

**Background:**

There are two types of voluntary interruption of pregnancy: elective and therapeutic abortion. These forms are different for many reasons, and it is reasonable to assume that they can have negative consequences that can last until a subsequent gestation. However, no study has analyzed the psychological experience of gestation after a previous abortion, distinguishing the two forms of voluntary interruption of pregnancy.

**Objective:**

This study aims to explore the level of prenatal attachment and centrality of pregnancy in nulliparous low-risk pregnant women with a recently (<3 years) previous elective or therapeutic abortion.

**Methods:**

A total of 34 nulliparous pregnant women with a history of abortion (23 elective and 11 therapeutic abortion), aged from 27 to 48 years (mean = 37.17), were recruited in the maternity ward of a public hospital of the metropolitan area of Tuscany and Lombardy (Italy) during the third trimester of gestation. The participants filled out a battery of questionnaires aimed at assessing prenatal attachment and centrality of pregnancy.

**Results:**

Analyses of variance showed that women with a history of elective abortion reported a higher centrality of pregnancy than women with a past therapeutic abortion. On the contrary, women with a past therapeutic abortion reported higher prenatal attachment.

**Conclusion:**

Elective and therapeutic abortions are different experiences that impact the way women experience a subsequent pregnancy. Future research should further investigate the psychological experience of gestation after abortion.

## Introduction

For a woman, the first gestation has been identified as a central life event (Darvill et al., 2010). From a psychological perspective, pregnancy and childbirth with the first baby involves the transition to motherhood, a major developmental period with important implications for mothers, for the infant–mother relationship, and for the infant’s development.

Overall, becoming a mother can be considered a turning point, during which a woman’s life takes a new direction, bringing many changes (Salmela-Aro et al., 2000). However, the way women live throughout gestation is linked to previous experiences, including termination of pregnancy. Several studies have analyzed the impact of spontaneous termination of pregnancy on subsequent gestations, revealing that a past miscarriage can lead to emotional and psychological distress for women (Côté-Arsenault et al., 2006; Tsartsara and Johnson, 2006; DeBackere et al., 2008; Smorti et al., 2020b). However, miscarriage is not the only loss of pregnancy; there are also voluntary terminations of pregnancy. To our knowledge, no studies have analyzed how a past voluntary termination of pregnancy is related to the psychological experience of subsequent gestation. This article aims to analyze the link between previous voluntary termination of pregnancy and subsequent gestation in nulliparous women.

Voluntary termination of pregnancy is not a unitary construct, given that it includes both elective and therapeutic abortions. These conditions are significantly different. Typically, elective abortion is defined as the procedure done in response to the mother’s choice (without a medical condition). This distinguishes it from therapeutic abortion, a procedure induced for medical reasons, such as to protect the mother’s health. Although a great debate exists about the fact that terminology and labels are extremely linked to moral judgment [“medical vs. elective is code for morally justified vs. morally unjustified (Janiak and Goldberg, 2016), as decided by someone other than the patient and her physician” (Watson, 2018, p. 1177)], in the absence of a new terminology, we choose to maintain “elective abortion” to define the pregnancy interruption for reasons other than medical.

In Italy, elective abortion can be performed within the first 90 days after conception and regards mainly unintended pregnancies (Torres and Forrest, 1988; Henshaw, 1998). Conversely, therapeutic abortion can be performed after the first 90 days and within 180 days of conception only if (a) the pregnancy or the birth is at a very high level of danger for the woman’s health or (b) there are established disease processes—including those related to relevant anomalies or malformations of the fetus—which constitute a grave danger to the physical or mental health of the woman. Given the terms of law in Italy, compared to other European countries, many women travel to England, the Netherlands, and Spain, where abortions are permitted until relatively late in pregnancy.

Given the different gestational ages, the intended termination of pregnancy may assume different characteristics, especially in terms of relation with fetus. Women who choose therapeutic abortion according to Italian law, compared to those who choose elective interruption, have usually seen an image of the fetus *via* ultrasound and have felt fetal movement, thus developing an image of the child-to-be. Seeing the fetus through ultrasound examinations, which increases the ability to differentiate and mentally imagine the fetus (Ji et al., 2005), allows the woman to enrich the image of her infant (Stern, 1988) with positive consequences on prenatal attachment (Sedgmen et al., 2006). On the other hand, with the perception of the first fetal movements by the beginning of the second trimester, the pregnant woman starts a process of psychological separation from the fetus that may help her to develop a prenatal attachment (Zeanah et al., 1990). With the intensification of fetal movement (Zeanah et al., 1990) and with the increase of gestational age, the maternal–fetal attachment becomes more affectionate and intense (Teixeira et al., 2016).

Women who develop prenatal attachment may experience an internal conflict when choosing abortion and emotion of loss toward the fetus (Major et al., 2000; Coleman et al., 2005) with psychological implications. Thus, the voluntary interruption of pregnancy during the second or third trimester seems to be related to a stronger maternal attachment to the fetus, with a potential negative impact on maternal mental health, which increases the risk of post-abortion post-traumatic stress disorder (Coleman et al., 2010). The negative impact of this experience could also be due to the procedure for therapeutic interruption of pregnancy, which involves labor induction abortion or, alternatively, surgical abortion, with increased pain and riskier treatment, which may be more stressful for women (Kersting et al., 2007).

Elective abortion constitutes a different experience for women, given that it is usually performed due to an unintended pregnancy. As these abortions are performed in the first trimester in Italy, women usually have not seen an image of the fetus in an ultrasound exam (Ji et al., 2005), have not felt fetal movements, and therefore probably have not developed a representation of the fetus as separate from themselves (Stern, 1988) and thus have not developed a strong attachment bond toward the fetus (Zeanah et al., 1990; Sedgmen et al., 2006).

Although an ultrasound exam is suggested to assure appropriate dating of the pregnancy prior to the execution of elective abortion procedure, women do not always see the image of the fetus. The pharmacological (drug-induced abortion) procedure or the surgical technique (through aspiration) used, far different from the labor induction performed for therapeutic abortion, may lead women to be less active participants.

To summarize, in Italy, a therapeutic abortion of a desired pregnancy may have very different implications for mental health than an elective abortion of an unintended pregnancy. To our knowledge, only one study has analyzed the psychological impact in elective and therapeutic abortions according to Italian law (Anselmi et al., 2018), highlighting a reduction of anxiety and depression 3 months after the procedure compared to the pre-procedure level (Anselmi et al., 2018). However, the limited sample (reduced by 65% at follow-up), the short follow-up (3 months), and the use of a self-report scale that measured psychopathological symptoms in relation to the previous week do not allow an understanding of the impact of elective or therapeutic abortion over time (Anselmi et al., 2018).

Studies conducted in different countries have shown that, while voluntary abortion, particularly in the first trimester of pregnancy, does not pose a substantial mental health risk in the short nor in the long term (Major et al., 2000; Steinberg et al., 2018), pregnancy termination for fetal abnormality in the second or third trimester increases women’s risk of post-traumatic stress disorder (PTSD) symptoms that can last up to 7 years after the abortion (Kersting et al., 2004; Korenromp et al., 2005). Despite the impact of this, most nulliparous women who had an abortion or a termination of pregnancy decided to have another child soon afterward (Rose et al., 2014), which means that psychological consequences from the previous loss may extend into the subsequent pregnancy. The psychological burden of a pregnancy after an abortion has not received much attention in literature, although it seems relevant. In fact, it would be important to understand how a past interruption of pregnancy could be linked to the centrality of subsequent gestation in terms of a turning point for the construction of a woman’s identity and to the quality of prenatal attachment. These aspects are strictly related to women’s psychological adjustment during pregnancy and post-partum and to the quality of maternal bond to the newborn (Tani et al., 2018; Ionio et al., 2019; Smorti et al., 2019, 2020a).

A literature review of the psychological effects of abortion has highlighted the controversy of two different perspectives: one is inclined to emphasize the risks for maternal mental health associated with abortion, while the other emphasizes pre-existing risk factors and minimizes the negative psychological effects (Reardon, 2018). The results showed that women who undergo second- and third-trimester abortions present significantly higher rates of post-traumatic stress symptoms than their peers who experience first-trimester abortions (Coleman et al., 2010). Moreover, women who decide to terminate their pregnancy due to fetal anomalies express sadness over the fetal loss (Lafarge et al., 2014), intense grief reaction, and psychological distress (Lohr et al., 2008). However, the psychological reactions are similar to those experienced by women after other prenatal losses, and there is no evidence that an abortion harms women’s health (American Psychological Association [APA], 2008). It has been suggested that conclusions regarding the psychological effects of voluntary termination of pregnancy are difficult, also due to the methodological limitations derived by the inclusion criteria—all women vs. women aged > 18 years, not controlling for parity (nulliparous vs. multiparous), typology of pregnancy termination (only induced termination or induced and spontaneous termination), and the reason of abortion (therapeutic vs. elective) (Reardon, 2018).

For this reason, considering the clinical and social relevance of these issues, research should be characterized by stricter inclusion criteria. For example, to our knowledge, no study has analyzed the psychological adaptation to gestation in nulliparous women after voluntary termination of pregnancy. This seems to be a relevant aspect, given that the way a woman experiences gestation may be affected by previous experiences, including the past termination of pregnancy. In particular, when the interruption of pregnancy is due to therapeutic reasons, it is reasonable to suppose that pregnancy may be lived as a more stressful experience.

## The Present Study

The main purpose of this study was to explore the centrality of pregnancy and the attachment bond to the unborn child in pregnant nulliparous women after a previous abortion experience, distinguishing between elective and therapeutic abortions.

We believed that therapeutic abortion, given its characteristics, constituted a greater risk factor for women’s psychological health than elective abortion, with a negative impact on the subsequent pregnancy adaptation. However, given the lack of studies about the impact of previous abortions on women’s actual gestation experience, this study has an explorative and descriptive aim, and no hypotheses are formulated.

## Methods

### Participants

Women were recruited for participation while waiting for a routine medical visit during the third trimester of pregnancy in the maternity ward of a public hospital of the metropolitan area of Tuscany and Lombardy (Italy). The inclusion criteria for participation in this study were as follows: (a) age > 18 years old, (b) able to speak and read Italian, (c) currently pregnant at > 32 weeks of gestation, (d) and had a voluntary termination of pregnancy in the past 3 years. The exclusion criteria for both groups were as follows: (a) history of miscarriage, (b) twin pregnancy, (c) fetal pathologies, and (d) maternal pathologies occurring before and/or during pregnancy. This information was obtained from the women’s medical records. The recruitment took place between October 2018 and September 2019. A psychologist responsible for the study approached women in the waiting room of the hospital and, after informing them about the study, asked for voluntary participation. Women who signed the informed consent received a self-report questionnaire. Only completed questionnaires were accepted for this study. Participation was voluntary, and no monetary reward was given.

Only 38 women who met the inclusion criteria were invited to participate. Nearly all (34, 89%) of the women who were invited consented to participate in the survey; four women (11%) refused without giving an explanation or by citing lack of time as the reason. The final sample of this study is constituted by 34 pregnant women aged from 27 to 48 (*M* = 37.17; *SD* = 5.87). Among them, 23 (67.6%) previously had an elective abortion, and 11 (32.4%) had a therapeutic abortion. All women had had only one elective or therapeutic abortion. All women (100%) were from Italy and of middle-high socioeconomic level; 91.1% had a high school diploma or university degree, and 76.4% had a job. Regarding marital status, 100% of the participants lived with their partners. None of the women reported having undergone a psychotherapeutic treatment following the abortion.

### Measures

Information about nationality (“Please indicate your place of birth”), education (“Please indicate your educational qualification”), work (“Please indicate your work condition”), and marital status (“Please indicate your romantic relationship situation”) were collected.

The Italian version of the Centrality of Events Scale (CES; Berntsen and Rubin, 2006; Ionio et al., 2019) was used. The Italian version of the CES is a self-report questionnaire specifically adapted to measure how pregnancy is central to an individual’s identity. It is composed of 20 items rated on a five-point Likert scale, ranging from 1 (totally disagree) to 5 (totally agree). In particular, the CES assesses three dimensions related to pregnancy: as a turning point in life (six items), a component of personal identity (six items), and an attribution of meaning to other personal life events (eight items). For each subscale, the total score is the sum of the items, with higher scores indicating more centrality on the experience of pregnancy. In our sample, the alpha values were 0.85, 0.84, and 0.81 for the three dimensions, respectively.

The Italian version of the Prenatal Attachment Inventory (PAI) (Müller, 1993; Busonera et al., 2017) was used to assess the maternal prenatal attachment to the unborn child. The PAI is constituted of 21 items rated on a four-point Likert scale, from 1 (almost never) to 4 (almost always). This self-report questionnaire measures four dimensions of the mother’s attachment bond to her child during pregnancy: differentiation of self from the fetus, affection, fantasy, sensitivity, and interaction. For each subscale, the total score is the sum of the items, with higher scores corresponding to a greater affection attachment to the newborn. For the present study, the values of Cronbach’s alpha ranged from 0.68 to 0.84 (0.68 for differentiation of self from the fetus, 0.72 for affection, 0.84 for fantasy, 0.68 for sensitivity, and 0.75 for interaction dimension).

### Procedure

The study was conducted in accordance with the guidelines for the ethical treatment of human participants of the Italian Psychological Association, and the procedures were approved by the ethics committee.

### Data Analysis

Data were analyzed using the Statistical Package for Social Sciences (SPSS), version 24 (2017). First, the normality of each variable was examined following the criteria indicated by Curran et al. (1996), according to which a range of ± 2 for skewness and ± 7 for kurtosis is considered to reflect a normal distribution of data. To compare the two groups, a series of independent *t*-tests, with group as independent variable and the dimensions of the CES and the PAI as dependent variables, was conducted. The alpha level was set to *p* = 0.05 for all tests, with confidence interval at 95%.

## Results

All variables present a normal distribution, with skewness values that ranged from −0.70 to 0.52 and kurtoses values that ranged from −1.18 to 1.26.

The socio-demographic characteristics of the two groups are reported in Table 1. No significant differences were found between women with a history of previous elective and therapeutic abortion, respectively, with respect to mean, educational level, and occupation status.

**TABLE 1:** Socio-demographic characteristics of the two groups.

	**Women with a previous elective abortion**	**Women with a previous therapeutic abortion**			

	***n* = 23**	***n* = 11**	**test**	**gdl**	***p***
**Age** M (SD)	36.04 (5.88)	39.54 (5.33)	*t* = −1.67	32	0.104
**Educational status n (%)**		Fisher’s exact = 1.23			0.732
Middle school	3 (13%)	–			
High school diploma	13 (56.5%)	7 (63.6%)			
Bachelor’s degree	7 (30.5%)	4 (36.4%)			
**Occupation status n (%)**		Fisher’s exact = 4.19			0.267
Housewife	1 (3.4%)	3 (27.3%)			
Employee	18 (78.3%)	8 (72.7%)			
Unemployed	2 (8.7%)	–			
Student	2 (8.7%)	–			

The results of the independent *t-tests* showed significant differences between the two groups. In particular, women with a history of previous elective abortion reported higher levels on all dimensions of the CES than women with a history of previous therapeutic abortion. On the contrary, women with a history of previous therapeutic abortion reported higher levels of prenatal attachment, referring to affection, fantasy, and the sensitivity dimensions of the PAI. The mean, standard deviation, and results of the independent *t-tests* are reported in Table 2.

**TABLE 2:** Means and standard deviations of the dimensions of the CES and the PAI in women with a history of previous elective and therapeutic abortion and independent *t*-tests results.

	**Women with a previous elective abortion**		**Women with a previous therapeutic abortion**					

	**(*n* = 23)**		**(*n* = 11)**					

	***M***	***SD***	***M***	***SD***	***t*(32)**	***p***	**95% CI**	
Pregnancy as a turning point in life (CES)	20.3	4.42	16.45	3.8	2.48	0.019	0.68	7.02
Pregnancy as a component of personal identity (CES)	31.87	4.42	26.46	5.87	3	0.005	1.74	9.09
Pregnancy as an attribution of meaning to other personal life events (CES)	20.65	4.95	16.64	4.5	2.27	0.03	0.48	7.55
Differentiation of self from the fetus (PAI)	12.48	2.35	13.54	1.97	−1.3	0.203	−2.74	0.6
Affection (PAI)	16.39	2.46	18.45	2.11	−2.38	0.023	−3.82	−0.3
Fantasy (PAI)	5.65	2.64	8.09	2.07	−2.44	0.011	−4.29	−0.59
Sensitivity (PAI)	12	2.59	14.27	1.74	−2.63	0.013	−4.03	−0.51
Interaction (PAI)	13.74	3.39	15.82	3.37	−1.68	0.103	−4.6	0.45

## Discussion

The aim of the present study was to analyze the centrality of pregnancy and the attachment bond to the unborn child in nulliparous women who were pregnant after previous voluntary interruption of gestation, distinguishing between elective and therapeutic abortion according to Italian law. Our results showed that women with a past elective abortion presented a higher centrality of pregnancy than women with a therapeutic abortion. In other words, it seems that pregnancy after an elective abortion is experienced as a major life event that constitutes a turning point compared to that experienced after a therapeutic abortion. This could be explained by considering that women who chose to interrupt their unintended pregnancy may express a higher awareness about the meaning of this gestation and about the wish to become a mother. Although pregnancy could be central also in women after a previous therapeutic abortion, our results highlighted that it constituted a turning point, especially for those who had had an elective abortion, as defined by Italian law.

Regarding prenatal attachment, our results showed that women with a past therapeutic abortion developed a more affectionate bond to the fetus during gestation. A possibility was that women with a past therapeutic abortion might use a more detached attitude toward the fetus due to the past loss. These data could have been explained by literature showing that a therapeutic abortion is experienced by women as a traumatic event (Kersting et al., 2004; Korenromp et al., 2005), which can lead to PTSD (Coleman et al., 2010), and that perinatal PTSD places women at risk for decreased maternal–infant attachment (Rogal et al., 2007). Despite this, our results highlighted a different scenario, with women who had had a therapeutic abortion reporting a higher prenatal attachment toward the unborn child in the third trimester of pregnancy than women with an elective abortion. This result could be interpreted considering previous studies that showed that pregnant women, after a previous perinatal loss, expressed higher levels of pregnancy-specific anxiety, which decreases as the current pregnancy progresses (Côté-Arsenault et al., 2006; Tsartsara and Johnson, 2006; Smorti et al., 2020b). Thus, it is reasonable to consider that, if during the first period of pregnancy women after a previous therapeutic abortion may be more cautious and concerned about gestation and fetus health, having passed the gestational age of the previous abortion, they can address their emotional attention to the unborn child, favoring a higher prenatal attachment than those who had had an elective abortion.

Considering the explorative nature of the present study, our results constitute a primary understanding of some aspects related to the psychological adjustment of pregnant women after an elective and a therapeutic interruption of pregnancy. In fact, given the lack of literature regarding this topic, our interpretation must be confirmed in further studies. In any case, the present study shows that gestation, after an interruption of pregnancy, is experienced in a different way from nulliparous women with a recent therapeutic or elective abortion and therefore implies that future research on the psychological well-being of pregnant women should take into consideration the different forms of abortion reported in clinical history.

The present study has some limitations. First, the size of our sample was quite small. However, the limited population was strengthened by the homogeneity of the sample that exceeds previous limits as revealed by literature review (Major et al., 2000; Reardon, 2018) because it was composed of nulliparous women with low-risk pregnancy, who had a recent (<3 years) voluntary interruption of pregnancy (excluding those with concurrent past miscarriage). Despite the small sample size, the homogeneity of the sample constituted by low-risk gestation (which excludes twin pregnancy, pregnancy with fetal pathologies and/or maternal pathologies, and previous mental health issues) without a miscarriage experience allows the generalization of results to anon-clinical population of pregnant women who performed a recent therapeutic or elective abortion.

Another limitation is that the study focuses on a single detection time, while a longitudinal study during the three trimesters of pregnancy and between pre- and post-partum periods would be more complete. However, this is the first study, to our knowledge, on the pregnancy adaptation of nulliparous women with a recent abortion. Finally, the use of self-report measures could be improved by using additional measures, such as semi-structured interviews that investigate the meaning of the pregnancy after an abortion, also in terms of maternal bond with the child.

Moreover, we did not include women with no history of interruption of pregnancy. Although our aim was to compare women who had had a form of interruption of pregnancy, the inclusion of a control group might have helped us obtain a more complete picture of the situation.

Despite these limitations, the present study offers an important contribution to literature, emphasizing how the experience of abortion could affect the experience of a subsequent pregnancy. In particular, our results highlight the relevance, in studies that investigate the psychological consequences of an abortion, to distinguish the type of abortion, elective or therapeutic. These constitute very different experiences not only with respect to the modality of abortion or the time in which it occurs but also especially for the psychological consequences that they can have on women.

## Data Availability Statement

The raw data supporting the conclusions of this article will be made available by the authors, without undue reservation.

## Ethics Statement

The study was approved by the Institute Ethics Committees of Pisa and Bergamo Hospital (CEAVNO n. 12749/2018 for Hospital in Pisa, and 196/2016 for Hospital in Bergamo). The patients/participants provided their written informed consent to participate in this study.

## Author Contributions

MS mainly wrote the introduction of the article. LP performed the data analysis mainly wrote the data analysis and results of the article. LB mainly wrote the discussion of the article. EC gave substantial contribution to acquisition of data and collaborated in writing the introduction. CI revised critically the manuscript for important intellectual content. All authors contributed to the article and approved the submitted version.

## Conflict of Interest

The authors declare that the research was conducted in the absence of any commercial or financial relationships that could be construed as a potential conflict of interest.
